# Genetic susceptibility to obesity and diet intakes: association and interaction analyses in the Malmö Diet and Cancer Study

**DOI:** 10.1007/s12263-013-0352-8

**Published:** 2013-07-17

**Authors:** Gull Rukh, Emily Sonestedt, Olle Melander, Bo Hedblad, Elisabet Wirfält, Ulrika Ericson, Marju Orho-Melander

**Affiliations:** 1 Diabetes and Cardiovascular Disease, Genetic Epidemiology, Department of Clinical Sciences in Malmö, Clinical Research Centre, Lund University, 91:12, Jan Waldenströms gata 35, 205 02 Malmö, Sweden; 2 Hypertension and Cardiovascular Disease, Department of Clinical Sciences in Malmö, Lund University, Malmö, Sweden; 3 Cardiovascular Epidemiology, Department of Clinical Sciences in Malmö, Lund University, Malmö, Sweden; 4 Nutrition Epidemiology, Department of Clinical Sciences in Malmö, Lund University, Malmö, Sweden

**Keywords:** Obesity susceptibility loci, Fat mass, Fat-free mass, Gene–diet interactions, Macronutrients, Genetic risk score

## Abstract

Gene–environment interactions need to be studied to better understand the obesity. We aimed at determining whether genetic susceptibility to obesity associates with diet intake levels and whether diet intakes modify the genetic susceptibility. In 29,480 subjects of the population-based Malmö Diet and Cancer Study (MDCS), we first assessed association between 16 genome-wide association studies identified obesity-related single-nucleotide polymorphisms (SNPs) with body mass index (BMI) and associated traits. We then conducted association analyses between a genetic risk score (GRS) comprising of 13 replicated SNPs and the individual SNPs, and relative dietary intakes of fat, carbohydrates, protein, fiber and total energy intake, as well as interaction analyses on BMI and associated traits among 26,107 nondiabetic MDCS participants. GRS associated strongly with increased BMI (*P* = 3.6 × 10^−34^), fat mass (*P* = 6.3 × 10^−28^) and fat-free mass (*P* = 1.3 × 10^−24^). Higher GRS associated with lower total energy intake (*P* = 0.001) and higher intake of fiber (*P* = 2.3 × 10^−4^). No significant interactions were observed between GRS and the studied dietary intakes on BMI or related traits. Of the individual SNPs, after correcting for multiple comparisons, *NEGR1* rs2815752 associated with diet intakes and *BDNF* rs4923461 showed interaction with protein intake on BMI. In conclusion, our study does not provide evidence for a major role for macronutrient-, fiber- or total energy intake levels in modifying genetic susceptibility to obesity measured as GRS. However, our data suggest that the number of risk alleles as well as some of the individual obesity loci may have a role in regulation of food and energy intake and that some individual loci may interact with diet.

**Electronic supplementary material:**

The online version of this article (doi:10.1007/s12263-013-0352-8) contains supplementary material, which is available to authorized users.

## Introduction

Obesity is a globally increasing major health problem, and considerable evidence from twin, adoption and family studies demonstrates that around 40–70 % of population variation in body mass index (BMI) is accounted by genetic factors (Herrera and Lindgren 2010; Maes et al. 1997; Stunkard et al. 1990). However, the rapid rises in the prevalence of overweight and obesity worldwide argue against a solely genetic etiology. The risk is more likely determined by environmental changes and complex interactions between genetic and environmental factors.

Recently, genome-wide association studies (GWAS) have discovered a large number of genetic loci robustly associated with various obesity-related traits (Frayling et al. 2007; Loos et al. 2008; Meyre et al. 2009; Thorleifsson et al. 2009; Willer et al. 2009; Heid et al. 2010; Speliotes et al. 2010). However, the mechanisms by which the identified variants affect the risk of obesity are mostly unclear. Several studies of the fat mass and obesity-associated gene (*FTO*) have indicated that environmental factors such as diet composition and physical activity level may modify the genetic susceptibility to develop obesity (Andreasen et al. 2008; Li et al. 2012; Sonestedt et al. 2011; Sonestedt et al. 2009). Apart from those in *FTO*, several of the identified SNPs are located in or near genes clearly related to appetite regulation (Willer et al. 2009), and recently, five of the novel obesity loci (*SH2B1, KCTD15, MTCH2, NEGR1* and *BDNF*) were indicated to be associated with dietary macronutrient intake levels (Bauer et al. 2009). Another study reported that the obesity-associated risk variants may affect the pattern and content of food consumption (McCaffery et al. 2012). On the other hand, the MONICA/KORA study comprising of 12,462 individuals found no evidence of nutritional intake or energy expenditure as mediators of genetic variant-BMI association (Holzapfel et al. 2010).

Apart from the studies that looked for the association between individual obesity-associated SNPs and diet intakes, studies investigating interaction between genetic predisposition score and dietary intakes or other environmental factors are lacking. A couple of studies have actually investigated interactions between genetic predisposition score for obesity and physical activity (Li et al. 2010) and/or television watching (Qi et al. 2012b), but with the best of our knowledge, no study so far has investigated interaction between genetic risk score (GRS) of obesity SNPs and dietary intakes.

In this study, we wanted to clarify whether dietary factors play a role in modifying the genetic susceptibility to obesity. For this, we first tested association between 16 GWAS identified obesity susceptibility variants and body composition traits in the population-based Malmö Diet and Cancer Study cohort (MDCS). We then created a GRS of the replicated variants and investigated associations with BMI and related traits and with dietary intake levels of fats, carbohydrates, protein, fiber as well as intake of total energy. Further, we studied interaction between the diet intakes and GRS on BMI and related traits. Finally, in post hoc analyses, we studied interactions between the individual SNPs and macronutrient intakes on BMI and body composition.

## Subjects and methods

### Study population

The Malmö Diet and Cancer Study (MDCS) is a large population-based cohort of Southern Sweden comprising of men born between 1923 and 1945 and women born between 1923 and 1950 who were invited to a baseline examination during 1991–1996 (Berglund et al. 1993; Manjer et al. 2002). Mental incapacity and limited Swedish language skills were the only exclusion criteria. All participants visited the study center on two occasions. During the first visit, anthropometric measures were taken, blood samples were collected, and the participants were provided with the questionnaires and detailed instructions about the dietary data collection procedure. During the second visit, after nearly 10 days, trained dietary interviewers conducted individual interviews to complete the diet history and to check the correctness of completed questionnaires. The MDCS study protocols were approved by the ethics committee of Lund University. All participants provided written informed consent. Full details on recruitment of participants and study procedures have been described previously (Manjer et al. 2001). In total, 30,447 individuals participated in baseline examinations (1991–1996). After excluding individuals without DNA or other crucial basic phenotypic information (*n* = 58) or lacking genotype information for more than 40 % of the SNPs (*n* = 909), 29,480 individuals remained and constituted the study sample for the genetic analyses. For the dietary analyses, we further excluded individuals with incomplete dietary data (*n* = 2,258) and/or with diabetes at baseline (*n* = 1,115) and were left with 26,107 individuals. Diabetes at baseline was identified through the use of antidiabetic therapy or self-reported diabetes diagnosis.

### Anthropometric measures

Weight was measured in kilograms using a balance-beam scale with subjects wearing light clothes and no shoes, height was measured in centimeters using a fixed stadiometer, and waist circumference was measured in centimeters by taking measurements midway between the lowest rib margin and iliac crest. Total body FM and fat-free mass (FFM) were determined using the body composition analyzer (BIA 103; JRL Systems, Mt. Clemens, MI, USA), which employs bioelectric impedance principle. Obesity was defined according to the criteria set by World Health Organization, i.e., individuals with BMI ≥ 30 kg/m^2^ were considered obese and those with BMI ≥ 25 kg/m^2^ but <30 kg/m^2^ were considered overweight (World Health Organization 2000).

### Dietary assessment

A modified dietary history method specifically designed for the MDCS was used and is described in detail earlier (Wirfalt et al. 2002). Shortly, the dietary history method combined a 7-day menu book (covering cooked lunches, dinner meals and cold beverages), a 168-item dietary questionnaire (covering foods regularly consumed during the past year not covered by the menu book) and a 1-h interview (concerning food preparation methods and portion sizes of foods collected in the menu book). The coding routines of the dietary data were slightly altered in September 1994 in order to shorten the interview time. Although the change did not have any major influence on the ranking of individuals, a variable for diet assessment method version has been constructed and is used as a covariate when appropriate. The average daily intake of foods was calculated based on the information available in the menu book, the questionnaire and interview. Food intakes were converted to nutrient intake data using the MDC Food and Nutrient Database, which was specifically developed for the MDC study and originated from PC KOST2-93 of the Swedish National Food Administration. The relative validity of the dietary method had been evaluated with 18 days of weighted food records as the reference method. Energy-adjusted Pearson correlation coefficients in women and men, respectively, were 0.53/0.54 for protein, 0.69/0.64 for fat, 0.70/0.66 for carbohydrates and 0.69/0.74 for fiber (Riboli et al. 1997).

### Genotyping

DNA was extracted from whole blood samples using Qiagen Maxipreps (Qiagen, Valencia, CA, USA). We selected 16 single-nucleotide polymorphisms (SNPs) identified through recent GWAS showing significant associations with BMI and/or obesity (Meyre et al. 2009; Thorleifsson et al. 2009; Willer et al. 2009). Of these, six SNPs (*MC4R* rs17782313, *SH2B1* rs7498665, *GNPDA2* rs10938397, *BDNF* rs4923461, *NCR3*/*AIF1*/*BAT2* rs2844479 and *FTO* rs9939609) were genotyped by Taqman allelic discrimination assay-by-design method using an ABI 7900 PCR system (Applied Biosystems, Foster City, CA, USA). Six SNPs (*MTCH2* rs10838738, *BCDIN3D*/*FAIM2* rs7138803, *SEC16B*/*RASAL2* rs10913469, *TMEM18* rs6548238, *MAF* rs1424233 and *NPC1* rs1805081) were genotyped using a Sequenom iPLEX platform (Sequenom, San Diego, CA, USA). Each of the remaining four SNPs was genotyped by two different methods. Two SNPs (*NEGR1* rs2815752 and *PTER* rs10508503) were genotyped by Sequenom and Taqman. The *KCTD15*/*CHST8* rs29941 and *SFRS10* rs7647305 were genotyped by a KASPar allelic discrimination method (KBioscience, Hoddesdon, UK) (rs7647305 and rs29941) or by Taqman and Sequenom methods, respectively. Average successful genotype call rate was 98.4 %. All SNPs were in Hardy–Weinberg equilibrium with *P* > 0.0031 (Bonferroni correction for 16 independent tests at *α* = 0.05), as determined by a chi-square test.

### Genetic risk score calculation

The GRS comprising of 13 out of the 16 genotyped SNPs was calculated by using PLINK (version 1.05). Three of the SNPs (*AIF1* rs2844479, *PTER* rs10508503 and *MAF* rs1424233) were not replicated in our study and were therefore not included in the GRS. Individual genotypes were recoded as 0, 1 and 2 according to the number of alleles associating with higher BMI and/or risk of obesity for each SNP defined on the basis of robust observations in GWAS (Meyre et al. 2009; Thorleifsson et al. 2009; Willer et al. 2009).

### Statistical analysis

All statistical analyses were performed with SPSS version 20 (IBM Corp., Armonk, NY, USA). To normalize the distributions, all variables were logarithmically transformed. Assuming additive model and adjusting for age and sex, we used logistic regression to analyze association between each SNP or GRS with overweight and obesity and linear regression to analyze association between the SNPs or GRS and quantitative variables (height, weight, waist and hip circumference, FM, FFM, body fat percentage and dietary variables including total energy intake (kcal/day), percentage of energy [*E*%] from fat, carbohydrates and protein or according to fiber density [g/1,000 kcal]). The analyses of associations with dietary variables were adjusted for age, sex, season, diet assessment method version and total energy intake.

The associations between GRS and BMI, FM and FFM were evaluated in strata of population-specific quintiles of macronutrients (*E*%]) or fiber (g/1,000 kcal). Interactions were assessed by including diet quintiles, GRS (treated as continuous variables), or the individual SNPs, and a multiplicative term of them to the analysis. Categorized dietary variables were used in order to handle skewness of the variables and to minimize the influence of extreme and possibly less reliably reported intakes. As dietary habits and diet reporting may differ and body fat composition differs between males and females, in addition to the main analyses of the whole cohort, we additionally report all GRS × diet and SNP × diet interaction analyses separately in males and females using gender-specific diet quintiles. Finally, in sensitivity analyses, we excluded 5,053 (18.6 %) individuals identified as putative nonadequate reporters of energy intake (under and overreporters, hereafter called as misreporters). Such misreporters were identified by comparing total energy intake and expenditure, and this method is described in detail elsewhere (Mattisson et al. 2005).

Power calculations were performed using Quanto (Quanto version 1.2.4: http://hydra.usc.edu/gxe). We estimated that with the sample size of this study and assuming an average minor allele frequency of 0.32, we were able to detect an effect size of 0.17 BMI units for each additional risk allele in GRS. Moreover, with an estimated effect size of 0.028 for dietary variables and 0.11 for GRS, we were able to detect a gene × diet interaction of at least 0.022 on BMI with 80 % power at *α* level of 0.05.

A *P* value of <0.05 was considered significant in the analyses between the SNPs or the GRS and the BMI and body composition variables as well as in the interaction analyses between the GRS and diet variables on BMI and associated traits. In contrast, the *P* values from association analyses of the individual SNPs with the dietary intakes were corrected for multiple testing adjusting for the number of the analyzed SNPs (*n* = 16), but not with the number of diet variables due to them being highly correlated. Similarly, we corrected the secondary interaction analyses between the individual SNPs and dietary intakes on BMI and body composition variables with the number of the analyzed SNPs (*n* = 16), but not with the number of diet variables nor with the number of BMI and related traits due to high correlation between the analyzed diet variables as well as between the BMI-related traits. A *P* value of ≤0.003125 was considered significant in the analyses corrected for multiple testing.

## Results

The characteristics of the study participants are reported in Table 1. Of the 29,480 individuals, 40.2 % were overweight, 14.1 % were obese, and 4.2 % had diabetes at the time of baseline examinations during 1991–1996.

**Table 1:** Characteristics of the Malmö Diet and Cancer Study (MDCS) participants

Variables	All	Men	Women
*n*	29,480	11,754	17,726
Age (years)	58.0 ± 7.6	59.1 ± 7.0	57.3 ± 7.9
Height (cm)	168.6 ± 8.9	176.3 ± 6.7	163.5 ± 6.1
Weight (kg)	73.6 ± 13.8	81.9 ± 12.4	68.2 ± 11.8
BMI (kg/m^2^)	25.8 ± 4.1	26.3 ± 3.6	25.5 ± 4.3
Waist (cm)	84.4 ± 13.1	93.9 ± 10.3	78.1 ± 10.7
Hip (cm)	98.6 ± 8.9	99.4 ± 7.3	98.0 ± 9.7
Fat mass (kg)	19.8 ± 6.9	17.4 ± 6.2	21.5 ± 6.9
Fat-free mass (kg)	53.2 ± 11.1	63.9 ± 8.3	46.2 ± 5.7
Body fat (%)	26.8 ± 7.0	20.8 ± 5.1	30.8 ± 5.0
Overweight (%)	40.2	49.6	33.9
Obesity (%)	14.1	13.7	14.3
Diabetes (%)	4.2	5.7	3.3
Total energy intake (kcal/day)	2,275 ± 655	2,644 ± 683	2,035 ± 507
Carbohydrate intake (*E*%)	45.2 ± 6.1	44.7 ± 6.2	45.5 ± 6.0
Protein intake (*E*%)	15.8 ± 2.6	15.5 ± 2.5	16.0 ± 2.6
Fat intake (*E*%)	39.0 ± 6.1	39.8 ± 6.3	38.5 ± 6.0
Fiber density (g/1,000 kcal)	9.1 ± 2.8	8.3 ± 2.5	9.6 ± 2.8
Misreporters of energy^a^ (%)	18.6	15.6	20.5

Obese: BMI ≥ 30 kg/m^2^; overweight: 25 kg/m^2^ ≤ BMI < 30 kg/m^2^. Data are presented as mean ± SD, unless otherwise indicated

*BMI* body mass index

^a^Misreporters of energy are individuals identified to report a nonadequately low or high energy intake or physical activity level

### Association of the obesity susceptibility SNPs with obesity-related traits

Of the tested 16 SNPs, 14 were directionally consistent with the results reported in the original GWAS concerning association with BMI, waist or hip circumference, and 11 SNPs reached statistical significance with at least one of these traits (Table 2). Variants in/near *FTO* and *TMEM18* showed the largest effect sizes for all of the three continuous traits. Variants in/near *FTO*, *MC4R*, *FAIM2*, *SEC16B* and *AIF1* associated with greater height and 11 SNPs associated with increased weight (Table 2).

**Table 2:** Association of 16 BMI or obesity-associated variants with anthropometric measures in the Malmö Diet and Cancer Study

Chr	Gene or nearby gene	SNP (1/2)	BMI (kg/m^2^)		Height (cm)		Weight (kg)		Waist (cm)		Hip (cm)	
			*β* ± SE	*P*	*β* ± SE	*P*	*β* ± SE	*P*	*β* ± SE	*P*	*β* ± SE	*P*
16	*FTO*	rs9939609 (A/T)	0.32 ± 0.04	7.0 × 10^−20^	0.11 ± 0.06	0.039	0.99 ± 0.11	2.6 × 10^−21^	0.70 ± 0.09	3.1 × 10^−14^	0.62 ± 0.08	3.5 × 10^−16^
18	*MC4R*	rs17782313 (C/T)	0.13 ± 0.04	0.002	0.18 ± 0.06	0.004	0.53 ± 0.12	2.3 × 10^−5^	0.30 ± 0.11	0.005	0.29 ± 0.09	0.001
16	*SH2B1*	rs7498665 (G/A)	0.086 ± 0.04	0.009	0.019 ± 0.005	0.72	0.27 ± 0.11	0.009	0.30 ± 0.09	0.001	0.27 ± 0.08	2.6 × 10^−4^
4	*GNPDA2*	rs10938397 (G/A)	0.12 ± 0.03	0.001	−0.026 ± 0.05	0.64	0.29 ± 0.10	0.003	0.28 ± 0.09	0.001	0.22 ± 0.07	0.002
11	*MTCH2*	rs10838738 (G/A)	0.038 ± 0.03	0.26	−0.14 ± 0.05	0.014	−0.002 ± 0.10	0.95	0.15 ± 0.09	0.11	0.075 ± 0.08	0.32
1	*NEGR1*	rs2815752 (T/C)	0.15 ± 0.03	1.3 × 10^−5^	−0.27 ± 0.05	2.3 × 10^−7^	0.17 ± 0.10	0.091	0.30 ± 0.09	0.001	0.25 ± 0.07	4.6 × 10^−4^
3	*SFRS10*	rs7647305 (C/T)	0.11 ± 0.04	0.005	0.040 ± 0.07	0.55	0.37 ± 0.13	0.004	0.38 ± 0.11	0.001	0.21 ± 0.09	0.022
12	*BCDIN3D*/*FAIM2*	rs7138803 (A/G)	0.085 ± 0.03	0.017	0.16 ± 0.05	0.002	0.38 ± 0.10	2.8 × 10^−4^	0.26 ± 0.09	0.005	0.25 ± 0.07	0.001
1	*SEC16B*/*RASAL2*	rs10913469 (C/T)	0.18 ± 0.03	2.9 × 10^−5^	0.19 ± 0.06	0.003	0.66 ± 0.12	1.2 × 10^−7^	0.38 ± 0.11	0.001	0.36 ± 0.09	9.8 × 10^−5^
11	*BDNF*	rs4923461 (A/G)	0.14 ± 0.04	0.001	0.012 ± 0.07	0.84	0.40 ± 0.13	0.001	0.29 ± 0.11	0.008	0.25 ± 0.09	0.007
19	*KCTD15*/*CHST8*	rs29941 (C/T)	0.065 ± 0.04	0.063	−0.053 ± 0.05	0.35	0.14 ± 0.11	0.19	0.081 ± 0.09	0.40	0.084 ± 0.08	0.27
2	*TMEM18*	rs6548238 (C/T)	0.19 ± 0.04	1.8 × 10^−5^	0.021 ± 0.07	0.69	0.59 ± 0.13	2.9 × 10^−5^	0.53 ± 0.12	9.0 × 10^−6^	0.34 ± 0.10	0.001
6	*NCR3*/*AIF1*/*BAT2*	rs2844479 (T/G)	−0.039 ± 0.04	0.25	0.40 ± 0.05	1.1 × 10^−13^	0.26 ± 0.10	0.019	−0.037 ± 0.09	0.55	0.10 ± 0.08	0.20
10	*PTER locus*	rs10508503 (C/T)	−0.004 ± 0.06	0.95	−0.095 ± 0.09	0.36	−0.099 ± 0.10	0.72	0.038 ± 0.15	0.74	−0.036 ± 0.13	0.82
16	*MAF locus*	rs1424233 (A/G)	0.023 ± 0.03	0.34	−0.18 ± 0.05	4.8 × 10^−4^	−0.099 ± 0.10	0.48	0.028 ± 0.09	0.62	0.055 ± 0.07	0.34
18	*NPC1*	rs1805081 (A/G)	0.097 ± 0.03	0.002	0.003 ± 0.05	0.97	0.28 ± 0.10	0.004	0.23 ± 0.09	0.008	0.21 ± 0.07	0.002

All anthropometrics (logarithmically transformed variables) are adjusted for age and sex

*Chr* chromosome number, *SNP* single-nucleotide polymorphism, (*1*/*2*) 1 refers to the risk allele, 2 refers to other allele; BMI, body mass index

SNPs in the *FTO, GNPDA2, SEC16B, BDNF, TMEM18* and *NPC1* loci associated with increased risk of both overweight and obesity (Table 3). SNPs in *SH2B1*, *SFRS10* and *KCTD15* associated with increased risk of overweight, but not with obesity, while the risk alleles in *MC4R* and *NEGR1* associated with increased risk of obesity, but not with overweight (Table 3).

**Table 3:** Association of 16 BMI or obesity-associated variants with overweight and obesity among participants of Malmö Diet and Cancer Study

Gene or nearby gene	SNP (1/2)	Lean versus overweight		Lean versus obese	
		OR (95 % CI)	*P*	OR (95 % CI)	*P*
*FTO*	rs9939609 (A/T)	1.12 (1.08–1.16)	4.8 × 10^−9^	1.21 (1.14–1.27)	3.9 × 10^−12^
*MC4R*	rs17782313 (C/T)	1.03 (0.99–1.08)	0.14	1.09 (1.03–1.16)	0.004
*SH2B1*	rs7498665 (G/A)	1.06 (1.02–1.10)	0.005	1.05 (0.99–1.10)	0.091
*GNPDA2*	rs10938397 (G/A)	1.07 (1.03–1.11)	0.001	1.10 (1.05–1.16)	1.4 × 10^−4^
*MTCH2*	rs10838738 (G/A)	1.00 (0.97–1.04)	0.86	1.03 (0.98–1.09)	0.25
*NEGR1*	rs2815752 (T/C)	1.04 (1.00–1.08)	0.051	1.09 (1.03–1.14)	0.002
*SFRS10*	rs7647305 (C/T)	1.06 (1.01–1.11)	0.021	1.04 (0.98–1.11)	0.20
*BCDIN3D*/*FAIM2*	rs7138803 (A/G)	1.02 (0.98–1.05)	0.37	1.05 (0.99–1.10)	0.086
*SEC16B*/*RASAL2*	rs10913469 (C/T)	1.05 (1.00–1.10)	0.032	1.12 (1.05–1.19)	3.8 × 10^−4^
*BDNF*	rs4923461 (A/G)	1.06 (1.01–1.11)	0.012	1.08 (1.01–1.15)	0.026
*KCTD15*/*CHST8*	rs29941 (C/T)	1.05 (1.02–1.10)	0.006	1.04 (0.99–1.10)	0.13
*TMEM18*	rs6548238 (C/T)	1.06 (1.01–1.11)	0.027	1.16 (1.08–1.24)	2.2 × 10^−5^
*NCR3*/*AIF1*/*BAT2*	rs2844479 (T/G)	1.00 (0.96–1.03)	0.79	0.98 (0.93–1.03)	0.46
*PTER locus*	rs10508503 (C/T)	1.00 (0.94–1.06)	0.92	1.01 (0.92–1.10)	0.84
*MAF locus*	rs1424233 (A/G)	1.00 (0.96–1.03)	0.89	0.98 (0.93–1.03)	0.43
*NPC1*	rs1805081 (A/G)	1.06 (1.02–1.10)	0.001	1.09 (1.04–1.15)	0.001

Adjusted for age and sex

*BMI* body mass index, *SNP* single-nucleotide polymorphism, (*1*/*2*) 1 refers to risk allele, and 2 refers to other allele; lean: BMI < 25 kg/m^2^, overweight: 25 kg/m^2^ ≥ BMI < 30 kg/m^2^, obese: BMI ≥ 30 kg/m^2^, *OR* odds ratio, *95* *% CI* 95 % confidence interval

SNPs in *FTO, GNPDA2, SFRS10, TMEM18* and *NPC1* loci associated with increased FM, FFM and body fat percentage (BF%). *SH2B1* and *NEGR1* SNPs associated with increased FM and BF%, while the *MTCH2* variant associated only with increased BF%. Variants in *MC4R, FAIM2, SEC16B* and *BDNF* loci associated with both higher FM and FFM. The risk allele of the SNP in *AIF1* locus associated with increased FFM and decreased BF% (Table 4).

**Table 4:** Association of 16 BMI or obesity-associated variants with measures of body composition among participants of Malmö Diet and Cancer Study

Gene or nearby gene	SNP (1/2)	Body fat (%)		Fat mass (kg)		Fat-free mass (kg)	
		*β* ± SE	*P*	*β* ± SE	*P*	*β* ± SE	*P*
*FTO*	rs9939609 (A/T)	0.24 ± 0.04	4.3 × 10^−8^	0.47 ± 0.06	5.6 × 10^−16^	0.52 ± 0.06	1.8 × 10^−18^
*MC4R*	rs17782313 (C/T)	0.071 ± 0.05	0.076	0.19 ± 0.07	0.002	0.33 ± 0.07	3.5 × 10^−6^
*SH2B1*	rs7498665 (G/A)	0.14 ± 0.04	0.005	0.20 ± 0.06	0.001	0.098 ± 0.06	0.077
*GNPDA2*	rs10938397 (G/A)	0.11 ± 0.04	0.031	0.16 ± 0.06	0.005	0.13 ± 0.06	0.024
*MTCH2*	rs10838738 (G/A)	0.11 ± 0.04	0.006	0.094 ± 0.06	0.090	−0.087 ± 0.06	0.17
*NEGR1*	rs2815752 (T/C)	0.17 ± 0.04	3.7 × 10^−5^	0.18 ± 0.06	0.001	0.007 ± 0.06	0.88
*SFRS10*	rs7647305 (C/T)	0.12 ± 0.05	0.028	0.18 ± 0.07	0.006	0.19 ± 0.07	0.013
*BCDIN3D*/*FAIM2*	rs7138803 (A/G)	0.068 ± 0.04	0.18	0.17 ± 0.06	0.006	0.21 ± 0.06	2.2 × 10^−4^
*SEC16B*/*RASAL2*	rs10913469 (C/T)	0.076 ± 0.05	0.21	0.25 ± 0.07	0.001	0.43 ± 0.07	8.8 × 10^−10^
*BDNF*	rs4923461 (A/G)	0.065 ± 0.05	0.30	0.15 ± 0.07	0.021	0.25 ± 0.07	0.001
*KCTD15*/*CHST8*	rs29941 (C/T)	0.047 ± 0.04	0.37	0.076 ± 0.06	0.21	0.069 ± 0.06	0.24
*TMEM18*	rs6548238 (C/T)	0.11 ± 0.06	0.037	0.24 ± 0.07	0.001	0.33 ± 0.08	1.9 × 10^−5^
*NCR3*/*AIF1*/*BAT2*	rs2844479 (T/G)	−0.12 ± 0.04	0.003	−0.025 ± 0.06	0.56	0.29 ± 0.06	5.0 × 10^−6^
*PTER locus*	rs10508503 (C/T)	0.049 ± 0.07	0.35	0.016 ± 0.10	0.58	−0.10 ± 0.10	0.47
*MAF locus*	rs1424233 (A/G)	0.063 ± 0.04	0.21	0.006 ± 0.06	0.67	−0.11 ± 0.06	0.072
*NPC1*	rs1805081 (A/G)	0.091 ± 0.04	0.034	0.14 ± 0.06	0.005	0.14 ± 0.06	0.017

Adjusted for age and sex

*BMI* body mass index, *SNP* single-nucleotide polymorphism, (*1*/*2*) 1 refers to risk allele, and 2 refers to other allele

### Association of genetic risk score (GRS) with obesity-related traits

To examine the cumulative associated effect of the obesity susceptibility SNPs, a GRS comprising of 13 SNPs was created. Three SNPs (*AIF1* rs2844479, *PTER* rs10508503 and *MAF* rs1424233) were not replicated in our study and were therefore not included in the GRS. Of these, *AIF1* was originally reported as a weight locus and the *PTER* and *MAF* as loci for morbid obesity. The *AIF1* rs2844479 allele that associated with higher weight in the original GWAS was observed to associate with lower BMI in the present study, while the *PTER* and *MAF* SNPs did not associate with BMI or obesity in the present study.

Each additional BMI-increasing allele of GRS associated with a 0.12 kg/m^2^ (standard error [SE] 0.01, *P* = 3.6 × 10^−34^) increase in BMI, which corresponds to a 347 g increase in body weight per allele for a person 170 cm tall. GRS associated with weight, waist circumference and hip circumference but did not show any association with height (Table 5). In addition, comparing the individuals having a low number of risk alleles (GRS of ≤11) with those having a high number (GRS ≥16) indicated a difference of 2.2 kg of total weight, 1.2 kg of FM and 1.0 kg of FFM between the groups (Electronic Supplementary Material, Fig. 2).

**Table 5:** Association of genetic risk score (GRS) comprising of 13 obesity SNPs with anthropometric measures, body composition traits and obesity in the Malmö Diet and Cancer Study

Trait	*β* ± SE	*P*
BMI (kg/m^2^)	0.12 ± 0.01	3.6 × 10^−34^
Weight (kg)	0.35 ± 0.03	2.6 × 10^−31^
Height (cm)	0.007 ± 0.02	0.59
Waist (cm)	0.29 ± 0.03	2.5 × 10^−29^
Hip (cm)	0.24 ± 0.02	4.2 × 10^−29^
Body fat (%)	0.10 ± 0.01	2.4 × 10^−16^
Fat mass (kg)	0.18 ± 0.02	6.3 × 10^−28^
Fat-free mass (kg)	0.17 ± 0.02	1.3 × 10^−24^

	OR (95 % CI)	*P*
Overweight	1.05 (1.04–1.06)	1.4 × 10^−19^
Obesity	1.08 (1.06–1.10)	1.2 × 10^−22^

Adjusted for age and sex

*BMI* body mass index, *SNP* single-nucleotide polymorphism, overweight: 25 kg/m^2^ ≥ BMI < 30 kg/m^2^, obese: BMI ≥ 30 kg/m^2^, *OR* odds ratio, *95* *% CI* 95 % confidence interval

Each additional risk allele of GRS associated with 5 % increased odds of being overweight and 8 % increased odds of being obese (Table 5). We observed an increase in odds ratios for obesity at baseline with the increasing GRS against the group with a GRS of ≤11 which included 21 % of the study participants and was used as a reference group. In total, 18 % of all individuals had a GRS of ≥16 and they had a 1.38-fold higher risk of being overweight (95 % CI 1.27–1.49*, P* = 1.6 × 10^−14^) and 1.64-fold higher risk of being obese (95 % CI 1.47–1.84*, P* = 5.1 × 10^−18^) compared with the reference group (Electronic Supplementary Material, Fig. 3).

### Association of the obesity susceptibility SNPs and GRS with total energy intake and intake levels of macronutrients and fiber

When the 16 SNPs were tested for association with dietary intakes, the obesity risk alleles of four of them associated nominally significantly with lower total energy intake *(FTO*, *GNPDA2*, *NEGR1* and *NPC1*) and one (*AIF1*) with higher energy intake. Only the association with *FTO* remained significant after correction for multiple comparisons (*P* = 0.001). Four loci (*FTO*, *MTCH2*, *NEGR1* and *MAF*) associated nominally significantly with intake levels of at least one of the macronutrients, but only the associations with *NEGR1* locus remained significant after multiple comparisons. The obesity risk allele of the *NEGR1* SNP associated significantly with lower fat intake (*P* = 3.2 × 10^−5^) but with higher carbohydrate (*P* = 3.3 × 10^−5^) and fiber intakes (*P* = 1.1 × 10^−4^) (Electronic Supplementary Material, Table S3). In sensitivity analyses after excluding misreporters, the association of the obesity risk allele of *FTO* rs9939609 with total energy intake became nonsignificant (*P* = 0.083), but the associations between the obesity risk allele of *NEGR1* rs2815752 with fat (*P* = 5.5 × 10^−5^), carbohydrates (*P* = 1.8 × 10^−4^) and fiber (*P* = 4.0 × 10^−6^) intakes remained unchanged.

Mean total energy intake and intake levels of protein and fiber, but not of carbohydrates or fat, differed significantly across GRS groups. Individuals with higher GRS had on average lower total energy intake (*P* = 0.001) and higher intake of protein (*P* = 0.011) and fiber (*P* = 2.3 × 10^−4^) (Electronic Supplementary Material, Fig. 4). However, after further adjustment for BMI, the association of protein intake with GRS was no more significant (*P* = 0.25), while the associations with lower total energy intake and higher fiber intake by higher GRS were unchanged. In sensitivity analyses after excluding misreporters, the association of GRS with protein intake became nonsignificant (*P* = 0.11), but the associations with total energy (*P* = 0.019) and fiber intakes (*P* = 2.1 × 10^−4^) remained significant.

### Interaction between GRS and dietary intakes on obesity-related traits

**Fig. 1:**
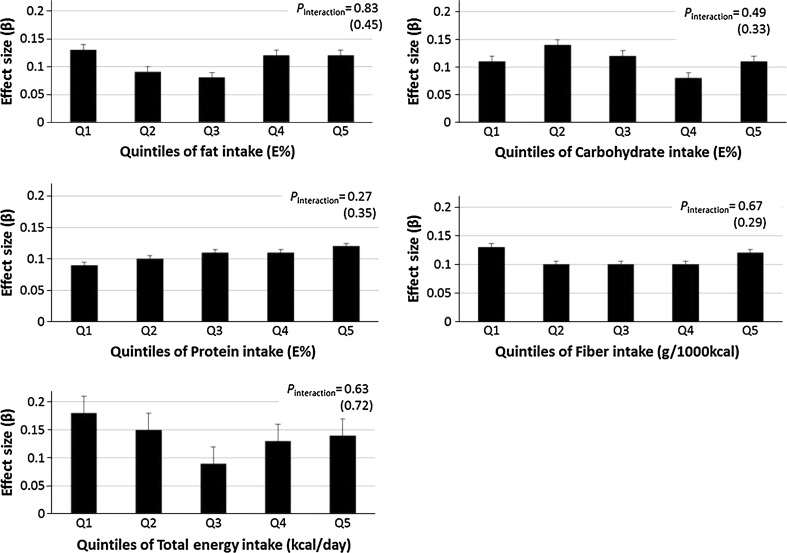
Associated effect sizes of the genetic risk score (GRS) on BMI according to intake quintiles of fat (*E*%), carbohydrate (*E*%), protein (*E*%), fiber (g/1,000 kcal) and total energy intake (kcal/day). *Error bars* represent standard error (SE). *P* values for interaction (*P*_interaction_) were adjusted for age, sex, season, diet assessment method version and total energy intake. The *P*_interaction_ values in *parenthesis* refer to sensitivity analyses after excluding misreporters

GRS was significantly associated with BMI in each diet and energy intake quintile with *P* values varying between *P* = 0.001 and *P* = 5.3 × 10^−10^ (Electronic Supplementary Material, Table S1a). However, the associated effect sizes did not differ significantly across the quintiles of fat (*P*_interaction_ = 0.82), carbohydrate (*P*_interaction_ = 0.49), protein (*P*_interaction_ = 0.27), fiber (*P*_interaction_ = 0.67) or total energy (*P*_interaction_ = 0.63) intakes (Fig. 1 and Electronic Supplementary Material, Table S1a). We also observed no significant interactions between GRS and dietary intakes on odds of being overweight or obese (Electronic Supplementary Material, Table S2a). Further adjustments with fiber intake or with physical activity levels did not affect any of the results, and results remained similar in sensitivity analysis by excluding potential misreporters.

In line with the BMI results, GRS associated with both FM and FFM in each diet quintile with *P* values varying between *P* = 0.017 and *P* = 1.7 × 10^−10^ (Electronic Supplementary Material, Table S1a), and no significant interactions were observed between GRS and quintiles of dietary or total energy intakes on neither FM nor FFM. The results remained similar in sensitivity analyses.

When repeating all analyses separately in both sexes, we did not observe any significant interactions between the diet variables and GRS on BMI, FM, FFM, overweight or obesity. All the results were similar using gender-specific diet quintiles as well as after excluding misreporters except that nominally significant interactions were observed between GRS and protein intake on obesity and FM among women in sensitivity analyses after excluding misreporters (*P*_interaction_ = 0.039 and 0.030, respectively) (Electronic Supplementary Material, Tables S1b, S1c, S2b and S2c).

### Interaction between the obesity susceptibility SNPs and dietary intakes on obesity-related traits

Of the studied SNPs, 11 indicated nominally significant interactions with at least one of the dietary intakes on BMI, FM or FFM (Electronic Supplementary Material, Table S4). However, after correction for multiple testing, only the interaction between the SNP rs4923461 in *BDNF* locus and protein intake on BMI remained significant (*P*_interaction_ = 0.001). Higher protein intake associated significantly with higher BMI in all *BDNF* genotype groups, but this association was significantly stronger among individuals with the obesity susceptibility allele A of *BDNF* rs4923461 (*P* = 1.0 × 10^−33^ and 1.1 × 10^−60^ among GA- and AA-genotype carriers, respectively) as compared to carriers of the GG-genotype (*P* = 2.7 × 10^−4^) (Electronic Supplementary Material, Fig. 5). In sensitivity analyses, after excluding misreporters, these associations remained similar (*P* = 1.1 × 10^−25^ and 1.3 × 10^−43^ for GA and AA-genotype carriers, respectively, as compared to *P* = 3.9 × 10^−4^ for GG-genotype carriers).

## Discussion

In our population-based cohort of 29,480 individuals, we replicated association between 13 of the 16 studied obesity susceptibility variants with BMI, overweight, obesity and body fat distribution. The SNPs at *AIF1*, *PTER* and *MAF* loci were not included in the GRS as they were not replicated in our study and have not been replicated in other studies either (den Hoed et al. 2012; Sandholt et al. 2011). When information of the 13 replicated SNPs was combined to GRS, each additional BMI-increasing allele associated with an average increase of 347 g of body weight. High GRS was found to associate with higher fiber intake but with lower total energy intake, but the dietary macronutrient, fiber or total energy intakes modified the association between GRS and BMI, FM or FFM, i.e., the associated obesogenic effects per allele of GRS did not significantly differ between the different diet intake groups. Analyses of individual SNPs revealed several nominally significant associations with dietary intakes and interactions with dietary intakes on BMI and associated traits of which association between the *NEGR1* locus and intake levels of fat, carbohydrates and fiber and interaction between the *BDNF* locus and protein intake levels remained significant after correction for multiple testing.

Genetic factors play an important role in obesity and its comorbidities. It is, however, not genes but environmental factors, i.e., increased energy intake and reduced energy expenditure that are considered primarily responsible for the worldwide rapid increase in obesity rate during the last decades (Hill et al. 2003; Slyper 2004). Consequently, better understanding of genetic and environmental determinants of body weight regulation and interactions between them can aid in determining the role of dietary habits in the prevalence and pathogenesis of obesity and be invaluable in facilitating identification of new tools to fight against the obesity epidemic.

It is evident that the studied BMI- and obesity-associated variants only explain a small proportion of heritability of obesity and population variance in BMI. Ignorance of complex pathways together with the scarcity of information on the role of rare variants and copy number variants may cover part of the unexplained heritability. Another potential source is gene–environment interactions. We hypothesized that the relative content of dietary fats, carbohydrates, protein or fiber could influence the genetic risk of being obese. However, our results suggest that the genetic associations between GRS and BMI and associated traits were not significantly modified by dietary macronutrient contents. A couple of studies have earlier reported interaction between an obesity SNP GRS and physical activity and/or television watching in relation to BMI. In these studies, GRS was composed of 12 and 32 BMI-associated SNPs, respectively (Li et al. 2010; Qi et al. 2012b). However, our study is the first to investigate the interaction between an obesity GRS and dietary intakes.

Majority of earlier studies have either been analyzing individual SNPs and their association with lifestyle factors, including diet intake levels or dietary patterns, or analyzed whether lifestyle factors mediate part of the association between the genetic risk variants and obesity (Holzapfel et al. 2010). We found nominal associations between total energy intake and five of the analyzed SNPs, but only the association with lower total energy intake and the obesity risk allele of *FTO* remained significant after correcting for multiple tests. However, in sensitivity analyses after excluding misreporters, the association of *FTO* with total energy intake became nonsignificant. In line with our findings, another study that investigated 12 obesity susceptibility SNPs among 1,700 European female participants did not find any significant associations between any of the studied SNPs and total energy intake (Bauer et al. 2009). We observed nominal associations between six of the obesity susceptibility SNPs and dietary intake levels of macronutrients, fiber or total energy intakes, and after correcting for multiple tests, the *NEGR1* locus remained significantly associated with lower fat, but with higher carbohydrate-and fiber intake levels. Similar to our results, Bauer et al. (2009) reported significant association of the *NEGR1* rs2568958 (proxy for rs2815752; *r* ^2^ = 1) with lower fat intake. Further, Lee et al. (2012) recently reported evidence for *NEGR1* being involved in body weight control and food intake in mice, which is in line with our results (Lee et al. 2012).

We did not find any significant interactions between GRS and total energy intake or macronutrient intakes on BMI or any of the associated traits, but we observed lower total energy intake and higher intake levels of protein and fiber among the individuals with higher GRS. As these associations could be secondary to the associations between GRS and BMI, or consequences of underreporting, we performed sensitivity analyses by excluding misreporters of energy as well as by additional adjustments for BMI and physical activity, but the associations of lower total energy intake and higher fiber intake with higher GRS remained significant in all these analyses. As the GRS, and risk alleles of four of the SNPs indicated association with lower total energy intake, it is possible that some of the obesity susceptibility variants could be involved in regulation of basal metabolic rate but more studies are needed to challenge this question. The question whether the association between higher GRS and higher fiber intake reflects involvement of fiber intake in appetite or weight regulation or the fact that overweight and obese individuals may eat differently remains unanswered in our study. However, additional adjustment with fiber intake did not influence the interaction between GRS and fats, carbohydrates or protein intakes on BMI, body composition or obesity.

The only significant interactions we detected were between GRS and protein intake on obesity and FM among women after excluding potential misreporters of energy intake. These interactions remained significant even after additionally adjusting for fiber intake and it would be interesting to see whether this finding would be replicated in other studies. Although we did not observe any significant interactions between GRS and dietary intakes on BMI or associated phenotypes, we observed a significant interaction between the *BDNF* locus and protein intake on BMI, the risk allele carriers having a stronger association between higher protein intake and higher BMI. We cannot exclude the possibility that some interactions may have left undetected due to insufficient power of our study to detect weaker interactions. Further, we need to emphasize that all the detected associations and the observed interaction between the *BDNF* locus and protein intake on BMI need to be confirmed in other studies to exclude by chance findings. Earlier studies have demonstrated that *BDNF* is involved in regulation of food intake, obesity and energy homeostasis (Rosas-Vargas et al. 2011). A recent study found that the association between *BDNF* rs6265 variant and obesity-related traits was modified by polyunsaturated fatty acid intakes among men in the Boston Puerto Rican population, which provides some further support for a role for *BDNF* in energy balance regulation and obesity (Ma et al. 2012). In addition to the interaction with *BDNF*, we observed several nominal interactions with some of the other susceptibility SNPs and diet, and we have earlier in MDCS reported interaction between dietary fat- and carbohydrate levels and the *FTO* genotype on FM (Sonestedt et al. 2011). Recently, a high-protein diet was found to be beneficial for weight loss and improvement of body composition and fat distribution among risk allele carriers of *FTO* variant rs1558902 evaluated in a 2-year diet intervention trial (Zhang et al. 2012). Another study conducted a randomized nutritional intervention with a Mediterranean-style-diet. After 3 years, the risk A-allele carriers of *FTO* rs9939609 variant had lower body weight gain than those without the risk allele. However, they did not find any significant interaction between nutritional intervention and the *FTO* variant (Razquin et al. 2010).

It is tempting to speculate that different obesity loci can partially act through different physiological mechanisms, and when the risk SNPs are simply combined to a GRS, the potential individual SNP interaction might get diluted or neutralized by interactions with specific dietary factors on specific directions. In addition, we only investigated interaction with intake levels of total energy, macronutrients and fiber and cannot exclude interaction with intake levels of other dietary factors like foods rich in carbohydrates or fats or with dietary patterns. Indeed, a recent study has reported that the genetic association of obesity SNP GRS with adiposity was more pronounced among individuals with greater consumption of sugar-sweetened beverages (Qi et al. 2012a). Obviously, further interaction and functional studies are required to understand the individual causal effects of these loci and variants.

The strength of our study is that MDCS is a large cohort with unique high-quality diet data as compared to most other epidemiological diet studies. A limitation of our study is the lack of BMI data at different time points so that we could follow the change during time. Secondly, so far at least 50 SNPs have been conclusively associated with obesity or related traits, but our study only included 16 SNPs which is another limitation of the current study. Furthermore, although our study is large and can be estimated to have sufficient power to detect relatively weak interactions, the evaluation of interactions between genotypes × dietary intakes on obesity is apparently difficult because of the complicated epidemiology of obesity and the very small effect sizes of the so far identified common genetic variants.

It is a generally accepted assumption that genetic and environmental factors that contribute to obesity interact in some way although very little is known about how such interactions take place. Our study suggests that higher susceptibility to obesity estimated as a GRS of 13 obesity-associated SNPs associates with lower total energy intake but with higher fiber intake and that some of the obesity loci like the *NEGR1* locus may regulate food intake. Further, we did not find evidence for a role of macronutrient, fiber or total energy intake levels in modifying the association between GRS and obesity traits although some individual obesity loci like the *BDNF* may interact with diet intakes. It is important that our results are replicated in other studies with dietary data of good quality and that more studies focus on the role and mechanisms of interactions between genes and diet in obesity.

## Electronic supplementary material

Below is the link to the electronic supplementary material.

Supplementary material 1 (DOC 559 kb)
