# Supplementary material for: Genetic susceptibility to obesity and diet intakes: association and interaction analyses in the Malmö Diet and Cancer Study
Source: Genes Nutr. 2013 Jul 17;8(6):535–47. doi: 10.1007/s12263-013-0352-8 (PMC3824829; doi:10.1007/s12263-013-0352-8)
Supplement: Supplementary file 1 — Supplementary material 1 (DOC 559 kb) [file 12263_2013_352_MOESM1_ESM.doc]

**SUPPLEMENTARY MATERIAL**

**Genetic susceptibility for obesity and diet intakes: association and interaction analyses in the Malmö Diet and Cancer Study**

**Rukh G^1^, Sonestedt E^1^, Melander O^2^, Hedblad B^3^, Wirfält E^4^, Ericson U^1^, Orho-Melander M^1^.**

**Correspondence to:**

Professor Marju Orho-Melander

Department of Clinical Sciences in Malmö

Lund University

Clinical research centre, 91:12, Jan Waldenströms gata 35

SE-205 02 Malmö

Sweden

Phone: +46 40 39 12 10

Fax: +46 40 39 12 22

E-mail: marju.orho-melander@med.lu.se

**Supplementary Tables**

**Table S1a.** Association between genetic risk score and BMI, fat mass and fat free mass in strata of dietary intakes among participants of the Malmö Diet and Cancer study

| **Tertiles** | **BMI** | | | **Fat mass** | | | **Fat free mass** | | |
| --- | --- | --- | --- | --- | --- | --- | --- | --- | --- |
|  | ***β* ± SE** | ***P*** | ***P*_interaction_** | ***β* ± SE** | ***P*** | ***P*_interaction_** | ***β* ± SE** | ***P*** | ***P*_interaction_** |
| **Fat (E%)** | | | | | | | | | |
| Quintile 1 | 0.13±0.02 | 1.1x10^-8^ | 0.82  (0.45) | 0.19±0.04 | 2.0x10^-7^ | 0.72  (0.42) | 0.19±0.04 | 9.5x10^-8^ | 0.97  (0.77) |
| Quintile 2 | 0.09±0.02 | 2.9x10^-5^ |  | 0.12±0.04 | 0.002 |  | 0.11±0.04 | 0.006 |  |
| Quintile 3 | 0.08±0.02 | 3.2x10^-4^ |  | 0.13±0.04 | 3.0x10^-4^ |  | 0.14±0.04 | 0.002 |  |
| Quintile 4 | 0.12±0.02 | 5.3x10^-8^ |  | 0.20±0.04 | 8.9x10^-8^ |  | 0.18±0.04 | 1.2x10^-6^ |  |
| Quintile 5 | 0.12±0.02 | 5.8x10^-8^ |  | 0.16±0.04 | 2.8x10^-5^ |  | 0.17±0.04 | 1.4x10^-5^ |  |
| **Carbohydrate (E%)** | | | | | | | | | |
| Quintile 1 | 0.11±0.02 | 1.2x10^-6^ | 0.49  (0.33) | 0.16±0.04 | 9.6x10^-6^ | 0.32  (0.14) | 0.13±0.04 | 0.001 | 0.56  (0.45) |
| Quintile 2 | 0.14±0.02 | 5.3x10^-10^ |  | 0.21±0.04 | 3.1x10^-8^ |  | 0.25±0.04 | 1.7x10^-10^ |  |
| Quintile 3 | 0.12±0.02 | 3.9x10^-7^ |  | 0.16±0.04 | 9.2x10^-6^ |  | 0.18±0.04 | 1.1x10^-5^ |  |
| Quintile 4 | 0.08±0.02 | 0.001 |  | 0.12±0.04 | 0.002 |  | 0.09±0.04 | 0.017 |  |
| Quintile 5 | 0.11±0.02 | 1.9x10^-7^ |  | 0.16±0.04 | 1.1x10^-5^ |  | 0.17±0.04 | 1.0x10^-5^ |  |
| **Protein (E%)** | | | | | | | | | |
| Quintile 1 | 0.09±0.02 | 1.1x10^-4^ | 0.27  (0.35) | 0.13±0.04 | 3.5x10^-4^ | 0.19  (0.26) | 0.12±0.04 | 0.006 | 0.22  (0.58) |
| Quintile 2 | 0.10±0.02 | 4.4x10^-6^ |  | 0.13±0.04 | 2.4x10^-4^ |  | 0.15±0.04 | 5.2x10^-5^ |  |
| Quintile 3 | 0.11±0.02 | 4.5x10^-7^ |  | 0.14±0.04 | 2.0x10^-4^ |  | 0.17±0.04 | 2.1x10^-5^ |  |
| Quintile 4 | 0.11±0.02 | 3.3x10^-7^ |  | 0.16±0.04 | 1.1x10^-5^ |  | 0.16±0.04 | 2.6x10^-6^ |  |
| Quintile 5 | 0.12±0.03 | 9.8x10^-8^ |  | 0.21±0.04 | 1.3x10^-8^ |  | 0.16±0.04 | 1.5x10^-5^ |  |
| **Fiber density (g/1000kcal)** | | | | | | | | | |
| Quintile 1 | 0.13±0.02 | 7.5x10^-9^ | 0.67  (0.29) | 0.18±0.04 | 3.6x10^-6^ | 0.78  (0.55) | 0.19±0.05 | 1.8x10^-5^ | 0.23  (0.24) |
| Quintile 2 | 0.10±0.02 | 1.3x10^-5^ |  | 0.12±0.04 | 0.001 |  | 0.21±0.04 | 2.2x10^-7^ |  |
| Quintile 3 | 0.10±0.02 | 7.3x10^-6^ |  | 0.14±0.04 | 3.8x10^-5^ |  | 0.15±0.04 | 8.0x10^-5^ |  |
| Quintile 4 | 0.10±0.02 | 1.7x10^-6^ |  | 0.17±0.04 | 1.0x10^-5^ |  | 0.11±0.04 | 0.002 |  |
| Quintile 5 | 0.12±0.02 | 3.5x10^-7^ |  | 0.20±0.04 | 1.4x10^-7^ |  | 0.15±0.04 | 8.2x10^-5^ |  |

Abbreviations: BMI, body mass index; E%, Energy %. Adjusted for age, sex, season, diet assessment method and total energy intake. *P*_interaction_ values after excluding misreporters are shown in parenthesis. Population specific diet quintiles were used. *P* and *P*_interaction_ values were calculated using logarithmically transformed

**Table S1b.** Association between genetic risk score and BMI, fat mass and fat free mass in strata of dietary intakes among male participants of the Malmö Diet and Cancer study

| **Tertiles** | **BMI** | | | **Fat mass** | | | **Fat free mass** | | |
| --- | --- | --- | --- | --- | --- | --- | --- | --- | --- |
|  | ***β* ± SE** | ***P*** | ***P*_interaction_** | ***β* ± SE** | ***P*** | ***P*_interaction_** | ***β* ± SE** | ***P*** | ***P*_interaction_** |
| **Fat (E%)** | | | | | | | | | |
| Quintile 1 | 0.12±0.03 | 5.4x10^-5^ | 0.77  (0.72) | 0.16±0.06 | 0.001 | 0.98  (0.85) | 0.15±0.07 | 0.025 | 0.54  (0.65) |
| Quintile 2 | 0.11±0.03 | 0.001 |  | 0.10±0.06 | 0.050 |  | 0.22±0.08 | 0.004 |  |
| Quintile 3 | 0.080±0.03 | 0.014 |  | 0.14±0.06 | 0.027 |  | 0.14±0.08 | 0.10 |  |
| Quintile 4 | 0.13±0.03 | 4.4x10^-5^ |  | 0.21±0.06 | 7.3x10^-5^ |  | 0.22±0.07 | 0.003 |  |
| Quintile 5 | 0.13±0.04 | 1.6x10^-4^ |  | 0.14±0.06 | 0.035 |  | 0.23±0.08 | 0.003 |  |
| **Carbohydrate (E%)** | | | | | | | | | |
| Quintile 1 | 0.12±0.03 | 4.2x10^-4^ | 0.48  (0.71) | 0.13±0.06 | 0.031 | 0.82  (0.78) | 0.17±0.08 | 0.031 | 0.39  (0.78) |
| Quintile 2 | 0.18±0.03 | 8.7x10^-8^ |  | 0.27±0.06 | 7.5x10^-6^ |  | 0.33±0.08 | 1.5x10^-5^ |  |
| Quintile 3 | 0.090±0.03 | 0.010 |  | 0.11±0.06 | 0.038 |  | 0.22±0.08 | 0.008 |  |
| Quintile 4 | 0.050±0.03 | 0.088 |  | 0.057±0.05 | 0.27 |  | 0.093±0.08 | 0.18 |  |
| Quintile 5 | 0.14±0.03 | 2.2x10^-6^ |  | 0.19±0.05 | 9.9x10^-5^ |  | 0.18±0.07 | 0.011 |  |
| **Protein (E%)** | | | | | | | | | |
| Quintile 1 | 0.13±0.03 | 5.4x10^-5^ | 0.87  (0.51) | 0.20±0.05 | 4.0x10^-4^ | 0.60  (0.76) | 0.21±0.08 | 0.009 | 0.76  (0.32) |
| Quintile 2 | 0.079±0.03 | 0.012 |  | 0.090±0.05 | 0.16 |  | 0.18±0.07 | 0.016 |  |
| Quintile 3 | 0.14±0.03 | 2.7x10^-6^ |  | 0.13±0.05 | 0.007 |  | 0.26±0.07 | 2.9x10^-4^ |  |
| Quintile 4 | 0.066±0.03 | 0.029 |  | 0.086±0.06 | 0.11 |  | 0.10±0.08 | 0.16 |  |
| Quintile 5 | 0.15±0.03 | 4.3x10^-6^ |  | 0.24±0.06 | 1.2x10^-5^ |  | 0.21±0.08 | 0.004 |  |
| **Fiber density (g/1000kcal)** | | | | | | | | | |
| Quintile 1 | 0.15±0.04 | 3.1x10^-5^ | 0.60  (0.64) | 0.18±0.06 | 0.002 | 0.61  (0.85) | 0.20±0.08 | 0.015 | 0.35  (0.76) |
| Quintile 2 | 0.092±0.03 | 0.005 |  | 0.075±0.05 | 0.24 |  | 0.24±0.08 | 0.002 |  |
| Quintile 3 | 0.12±0.03 | 1.1x10^-4^ |  | 0.14±0.06 | 0.003 |  | 0.28±0.07 | 2.2x10^-4^ |  |
| Quintile 4 | 0.090±0.03 | 0.004 |  | 0.15±0.06 | 0.004 |  | 0.071±0.08 | 0.35 |  |
| Quintile 5 | 0.13±0.03 | 2.4x10^-5^ |  | 0.21±0.05 | 2.3x10^-4^ |  | 0.18±0.07 | 0.008 |  |

Abbreviations: BMI, body mass index; E%, Energy %. Adjusted for age, season, diet assessment method and total energy intake. *P*_interaction_ values after excluding misreporters are shown in parenthesis. Gender specific diet quintiles were used. *P* and *P*_interaction_ values were calculated using logarithmically transformed variables.

**Table S1c.** Association between genetic risk score and BMI, fat mass and fat free mass in strata of dietary intakes among female participants of Malmö diet and cancer study

| **Tertiles** | **BMI** | | | **Fat mass** | | | **Fat free mass** | | |
| --- | --- | --- | --- | --- | --- | --- | --- | --- | --- |
|  | ***β* ± SE** | ***P*** | ***P*_interaction_** | ***β* ± SE** | ***P*** | ***P*_interaction_** | ***β* ± SE** | ***P*** | ***P*_interaction_** |
| **Fat (E%)** | | | | | | | | | |
| Quintile 1 | 0.12±0.03 | 1.0x10^-4^ | 0.80  (0.41) | 0.19±0.05 | 4.7x10^-4^ | 0.37  (0.15) | 0.20±0.04 | 4.0x10^-6^ | 0.75  (0.78) |
| Quintile 2 | 0.11±0.03 | 3.1x10^-4^ |  | 0.16±0.05 | 0.002 |  | 0.11±0.04 | 0.023 |  |
| Quintile 3 | 0.060±0.03 | 0.035 |  | 0.10±0.05 | 0.030 |  | 0.060±0.04 | 0.20 |  |
| Quintile 4 | 0.13±0.03 | 1.5x10^-5^ |  | 0.23±0.05 | 1.3x10^-5^ |  | 0.20±0.04 | 3.0x10^-6^ |  |
| Quintile 5 | 0.11±0.03 | 2.5x10^-4^ |  | 0.17±0.05 | 1.8x10^-4^ |  | 0.13±0.04 | 0.002 |  |
| **Carbohydrate (E%)** | | | | | | | | | |
| Quintile 1 | 0.11±0.03 | 2.5x10^-4^ | 0.62  (0.36) | 0.20±0.05 | 1.6x10^-5^ | 0.23  (0.080) | 0.10±0.04 | 0.014 | 0.90  (0.50) |
| Quintile 2 | 0.12±0.03 | 8.2x10^-5^ |  | 0.19±0.05 | 0.001 |  | 0.20±0.04 | 1.8x10^-6^ |  |
| Quintile 3 | 0.11±0.03 | 2.7x10^-4^ |  | 0.16±0.05 | 0.001 |  | 0.14±0.04 | 0.001 |  |
| Quintile 4 | 0.11±0.03 | 3.1x10^-4^ |  | 0.18±0.05 | 3.4x10^-4^ |  | 0.092±0.04 | 0.041 |  |
| Quintile 5 | 0.10±0.03 | 0.001 |  | 0.14±0.05 | 0.008 |  | 0.16±0.04 | 2.8x10^-4^ |  |
| **Protein (E%)** | | | | | | | | | |
| Quintile 1 | 0.049±0.03 | 0.010 | 0.11  (0.052) | 0.077±0.05 | 0.11 | 0.11  (0.039) | 0.056±0.04 | 0.21 | 0.056  (0.092) |
| Quintile 2 | 0.10±0.03 | 4.3x10^-4^ |  | 0.14±0.05 | 0.001 |  | 0.14±0.04 | 0.001 |  |
| Quintile 3 | 0.11±0.03 | 2.9x10^-4^ |  | 0.17±0.05 | 0.002 |  | 0.13±0.04 | 0.007 |  |
| Quintile 4 | 0.14±0.03 | 1.8x10^-6^ |  | 0.22±0.05 | 4.1x10^-6^ |  | 0.20±0.04 | 1.4x10^-6^ |  |
| Quintile 5 | 0.12±0.03 | 2.5x10^-4^ |  | 0.20±0.05 | 1.1x10^-4^ |  | 0.14±0.04 | 0.001 |  |
| **Fiber density (g/1000kcal)** | | | | | | | | | |
| Quintile 1 | 0.15±0.03 | 8.7x10^-7^ | 0.62  (0.24) | 0.26±0.05 | 8.2x10^-7^ | 0.55  (0.18) | 0.19±0.05 | 3.8x10^-5^ | 0.45  (0.23) |
| Quintile 2 | 0.077±0.03 | 0.015 |  | 0.11±0.05 | 0.033 |  | 0.14±0.04 | 0.001 |  |
| Quintile 3 | 0.045±0.03 | 0.11 |  | 0.064±0.05 | 0.14 |  | 0.060±0.04 | 0.13 |  |
| Quintile 4 | 0.17±0.03 | 9.2x10^-9^ |  | 0.27±0.05 | 2.9x10^-8^ |  | 0.19±0.04 | 9.1x10^-6^ |  |
| Quintile 5 | 0.092±0.03 | 0.003 |  | 0.15±0.05 | 0.004 |  | 0.11±0.04 | 0.011 |  |

Abbreviations: BMI, body mass index; E%, Energy %. Adjusted for age, season, diet assessment method and total energy intake. *P*_interaction_ values after excluding misreporters are shown in parenthesis. Gender specific diet quintiles were used. *P* and *P*_interaction_ values were calculated using logarithmically transformed variables.

**Table S2a.** Association between the genetic risk score and overweight and obesity in strata of dietary intakes among all participants of the Malmö Diet and Cancer study

| **Tertiles** | **Overweight** | | | **Obesity** | | |
| --- | --- | --- | --- | --- | --- | --- |
|  | **OR (95% CI)** | ***P*** | ***P*_interaction_** | **OR (95% CI)** | ***P*** | ***P*_interaction_** |
| **Fat (E%)** | | | | | | |
| Quintile 1 | 1.06(1.03-1.08) | 3.5x10^-5^ | 0.60  (0.42) | 1.08(1.05-1.12) | 1.1x10^-5^ | 0.61  (0.57) |
| Quintile 2 | 1.04(1.02-1.07) | 0.002 |  | 1.06(1.02-1.10) | 0.003 |  |
| Quintile 3 | 1.04(1.01-1.07) | 0.004 |  | 1.04(1.01-1.08) | 0.025 |  |
| Quintile 4 | 1.05(1.02-1.08) | 1.5x10^-4^ |  | 1.08(1.05-1.13) | 1.7x10^-5^ |  |
| Quintile 5 | 1.07(1.04-1.09) | 1.0x10^-6^ |  | 1.09(1.05-1.13) | 1.1x10^-5^ |  |
| **Carbohydrate (E%)** | | | | | | |
| Quintile 1 | 1.06(1.03-1.09) | 1.2x10^-5^ | 0.62  (0.53) | 1.07(1.03-1.11) | 2.8x10^-4^ | 0.49  (0.62) |
| Quintile 2 | 1.06(1.04-1.09) | 3.8x10^-6^ |  | 1.11(1.07-1.15) | 2.6x10^-8^ |  |
| Quintile 3 | 1.03(1.00-1.06) | 0.025 |  | 1.06(1.03-1.10) | 0.001 |  |
| Quintile 4 | 1.06(1.04-1.09) | 4.2x10^-6^ |  | 1.04(1.00-1.08) | 0.037 |  |
| Quintile 5 | 1.04(1.02-1.07) | 0.001 |  | 1.08(1.04-1.12) | 9.6x10^-5^ |  |
| **Protein (E%)** | | | | | | |
| Quintile 1 | 1.04(1.02-1.07) | 0.001 | 0.71  (0.90) | 1.07(1.03-1.12) | 0.001 | 0.39  (0.36) |
| Quintile 2 | 1.06(1.04-1.09) | 2.0x10^-6^ |  | 1.04(1.00-1.08) | 0.061 |  |
| Quintile 3 | 1.03(1.01-1.06) | 0.016 |  | 1.09(1.05-1.13) | 2.6x10^-5^ |  |
| Quintile 4 | 1.05(1.02-1.08) | 1.3x10^-4^ |  | 1.08(1.04-1.12) | 4.5x10^-5^ |  |
| Quintile 5 | 1.06(1.03-1.09) | 1.3x10^-5^ |  | 1.08(1.05-1.12) | 4.6x10^-6^ |  |
| **Fiber density (g/1000kcal)** | | | | | | |
| Quintile 1 | 1.06(1.03-1.08) | 5.9x10^-5^ | 0.69  (0.73) | 1.10(1.06-1.14) | 1.8x10^-6^ | 0.73  (0.21) |
| Quintile 2 | 1.06(1.04-1.09) | 3.6x10^-6^ |  | 1.07(1.03-1.11) | 0.001 |  |
| Quintile 3 | 1.04(1.02-1.07) | 0.001 |  | 1.05(1.01-1.09) | 0.010 |  |
| Quintile 4 | 1.03(1.01-1.06) | 0.017 |  | 1.07(1.03-1.11) | 1.9x10^-4^ |  |
| Quintile 5 | 1.06(1.04-1.09) | 4.8x10^-6^ |  | 1.08(1.04-1.12) | 2.8x10^-5^ |  |

Abbreviations: BMI, body mass index; E%, Energy %; Overweight: 25 kg/m^2^ ≥ BMI <30 kg/m^2^; Obesity: BMI ≥30 kg/m^2^; OR, odds ratio; 95% CI, 95% confidence interval. Adjusted for age, sex, season, diet assessment method and total energy intake. *P*_interaction_ values after excluding misreporters are shown in parenthesis. Population specific diet quintiles were used. *P* and *P*_interaction_ values were calculated using logarithmically transformed variables.

**Table S2b.** Association between the genetic risk score and overweight and obesity in strata of dietary intakes among male participants of Malmö Diet and Cancer study

| **Tertiles** | **Overweight** | | | **Obesity** | | |
| --- | --- | --- | --- | --- | --- | --- |
|  | **OR (95% CI)** | ***P*** | ***P*_interaction_** | **OR (95% CI)** | ***P*** | ***P*_interaction_** |
| **Fat (E%)** | | | | | | |
| Quintile 1 | 1.05(1.01-1.09) | 0.014 | 0.33  (0.23) | 1.08(1.02-1.15) | 0.014 | 0.67  (0.77) |
| Quintile 2 | 1.07(1.03-1.12) | 0.001 |  | 1.09(1.03-1.16) | 0.005 |  |
| Quintile 3 | 1.04(1.00-1.08) | 0.068 |  | 1.06(1.00-1.13) | 0.061 |  |
| Quintile 4 | 1.10(1.06-1.15) | 1.0x10^-6^ |  | 1.09(1.03-1.16) | 0.004 |  |
| Quintile 5 | 1.07(1.03-1.12) | 0.001 |  | 1.10(1.04-1.17) | 0.002 |  |
| **Carbohydrate (E%)** | | | | | | |
| Quintile 1 | 1.10(1.05-1.15) | 9.0x10^-6^ | 0.046  (0.086) | 1.08(1.01-1.14) | 0.017 | 0.74  (0.89) |
| Quintile 2 | 1.10(1.06-1.15) | 3.0x10^-6^ |  | 1.16(1.09-1.23) | 1.0x10^-6^ |  |
| Quintile 3 | 1.03(0.99-1.08) | 0.117 |  | 1.05(0.99-1.12) | 0.11 |  |
| Quintile 4 | 1.07(1.03-1.12) | 0.001 |  | 1.03(0.97-1.10) | 0.32 |  |
| Quintile 5 | 1.04(1.00-1.07) | 0.042 |  | 1.12(1.05-1.19) | 0.001 |  |
| **Protein (E%)** | | | | | | |
| Quintile 1 | 1.07(1.03-1.11) | 0.001 | 0.26  (0.64) | 1.11(1.04-1.19) | 0.001 | 0.88  (0.51) |
| Quintile 2 | 1.05(1.01-1.09) | 0.027 |  | 1.06(1.00-1.13) | 0.062 |  |
| Quintile 3 | 1.07(1.03-1.12) | 0.001 |  | 1.11(1.05-1.19) | 0.001 |  |
| Quintile 4 | 1.06(1.01-1.10) | 0.010 |  | 1.05(0.99-1.12) | 0.11 |  |
| Quintile 5 | 1.1(1.06-1.16) | 4.0x10^-6^ |  | 1.12(1.05-1.18) | 2.1x10^-4^ |  |
| **Fiber density (g/1000kcal)** | | | | | | |
| Quintile 1 | 1.07(1.02-1.11) | 0.002 | 0.27  (0.45) | 1.11(1.04-1.18) | 0.001 | 0.91  (0.66) |
| Quintile 2 | 1.09(1.04-1.13) | 4.8x10^-5^ |  | 1.08(1.01-1.14) | 0.019 |  |
| Quintile 3 | 1.10(1.06-1.15) | 6.0x10^-6^ |  | 1.08(1.02-1.15) | 0.015 |  |
| Quintile 4 | 1.03(0.99-1.08) | 0.099 |  | 1.06(1.00-1.13) | 0.052 |  |
| Quintile 5 | 1.06(1.02-1.10) | 0.006 |  | 1.11(1.04-1.17) | 0.001 |  |

Abbreviations: BMI, body mass index; E%, Energy %; Overweight: 25 kg/m^2^ ≥ BMI <30 kg/m^2^; Obesity: BMI ≥30 kg/m^2^; OR, odds ratio; 95% CI, 95% confidence interval. Adjusted for age, season, diet assessment method and total energy intake. *P*_interaction_ values after excluding misreporters are shown in parenthesis. Gender specific diet quintiles were used. *P* and *P*_interaction_ values were calculated using logarithmically transformed variables.

**Table S2c.** Association between the genetic risk score and overweight and obesity in strata of dietary intakes among female participants of Malmö Diet and Cancer study

| **Tertiles** | **Overweight** | | | **Obesity** | | |
| --- | --- | --- | --- | --- | --- | --- |
|  | **OR (95% CI)** | ***P*** | ***P*_interaction_** | **OR (95% CI)** | ***P*** | ***P*_interaction_** |
| **Fat (E%)** | | | | | | |
| Quintile 1 | 1.05(1.02-1.09) | 0.002 | 0.73  (0.85) | 1.07(1.03-1.12) | 0.002 | 0.85  (0.73) |
| Quintile 2 | 1.04(1.01-1.08) | 0.020 |  | 1.06(1.01-1.11) | 0.011 |  |
| Quintile 3 | 1.02(1.00-1.06) | 0.19 |  | 1.03(0.98-1.08) | 0.24 |  |
| Quintile 4 | 1.04(1.00-1.07) | 0.031 |  | 1.09(1.04-1.14) | 4.4x10^-4^ |  |
| Quintile 5 | 1.04(1.01-1.08) | 0.011 |  | 1.07(1.02-1.12) | 0.011 |  |
| **Carbohydrate (E%)** | | | | | | |
| Quintile 1 | 1.04(1.00-1.07) | 0.035 | 0.39  (0.45) | 1.07(1.02-1.12) | 0.005 | 0.46  (0.49) |
| Quintile 2 | 1.04(1.00-1.07) | 0.035 |  | 1.08(1.04-1.14) | 0.001 |  |
| Quintile 3 | 1.03(0.99-1.06) | 0.13 |  | 1.06(1.01-1.11) | 0.020 |  |
| Quintile 4 | 1.06(1.02-1.09) | 0.001 |  | 1.06(1.01-1.11) | 0.021 |  |
| Quintile 5 | 1.05(1.02-1.08) | 0.005 |  | 1.06(1.01-1.11) | 0.022 |  |
| **Protein (E%)** | | | | | | |
| Quintile 1 | 1.03(0.99-1.06) | 0.13 | 0.45  (0.38) | 1.03(0.98-1.09) | 0.26 | 0.076  (0.030) |
| Quintile 2 | 1.05(1.02-1.09) | 0.002 |  | 1.04(0.99-1.09) | 0.15 |  |
| Quintile 3 | 1.01(0.98-1.04) | 0.53 |  | 1.08(1.03-1.13) | 0.003 |  |
| Quintile 4 | 1.05(1.02-1.09) | 0.002 |  | 1.08(1.03-1.13) | 0.001 |  |
| Quintile 5 | 1.05(1.01-1.08) | 0.005 |  | 1.08(1.04-1.13) | 3.1x10^-4^ |  |
| **Fiber density (g/1000kcal)** | | | | | | |
| Quintile 1 | 1.04(1.01-1.08) | 0.013 | 0.20  (0.34) | 1.11(1.06-1.17) | 1.4x10^-5^ | 0.50  (0.26) |
| Quintile 2 | 1.03(0.99-1.06) | 0.14 |  | 1.04(0.99-1.09) | 0.093 |  |
| Quintile 3 | 1.01(0.97-1.04) | 0.77 |  | 1.01(0.97-1.06) | 0.62 |  |
| Quintile 4 | 1.06(1.03-1.10) | 4.3x10^-4^ |  | 1.11(1.06-1.17) | 1.0x10^-5^ |  |
| Quintile 5 | 1.06(1.03-1.10) | 3.2x10^-4^ |  | 1.05(1.01-1.10) | 0.031 |  |

Abbreviations: BMI, body mass index; E%, Energy %; Overweight: 25 kg/m^2^ ≥ BMI <30 kg/m^2^; Obesity: BMI ≥30 kg/m^2^; OR, odds ratio; 95% CI, 95% confidence interval. Adjusted for age, season, diet assessment method and total energy intake. *P*_interaction_ values after excluding misreporters are shown in parenthesis. Gender specific diet quintiles were used. *P* and *P*_interaction_ values were calculated using logarithmically transformed variables.

**Table S3.** Association of 16 obesity susceptibility SNPs with dietary intakes (as continuous variable) among non-diabetic participants of the Malmö Diet and Cancer Study

| Gene or nearby gene | SNP (1/2)^a^ | Total Energy (kcal/day) | | Fat (E%) | | Carbohydrate (E%) | | Protein (E%) | | Fiber (g/1000kcal) | |
| --- | --- | --- | --- | --- | --- | --- | --- | --- | --- | --- | --- |
|  |  | β | p-value | β | p-value | β | p-value | β | p-value | β | p-value |
| *FTO* | rs9939609 (A/T) | -17.62 | 0.001 | 0.017 | 0.69 | -0.070 | 0.22 | 0.053 | 0.011 | 0.062 | 0.004 |
| *MC4R* | rs17782313 (C/T) | 2.80 | 0.58 | 0.008 | 0.99 | -0.024 | 0.68 | 0.016 | 0.58 | 0.039 | 0.23 |
| *SH2B1* | rs7498665 (G/A) | 2.81 | 0.85 | 0.046 | 0.49 | -0.013 | 0.80 | -0.033 | 0.079 | -0.018 | 0.41 |
| *GNPDA2* | rs10938397 (G/A) | -10.97 | 0.039 | -0.016 | 0.84 | 0.019 | 0.70 | -0.003 | 0.89 | -0.011 | 0.68 |
| *MTCH2* | rs10838738 (G/A) | -5.00 | 0.41 | -0.030 | 0.57 | 0.008 | 0.75 | 0.022 | 0.32 | 0.057 | 0.009 |
| *NEGRI* | rs2815752 (T/C) | -12.15 | 0.004 | -0.21 | 3.2x10^-5^ | 0.23 | 3.3x10^-5^ | -0.022 | 0.26 | 0.084 | 1.1x10^-4^ |
| *SFRS10* | rs7647305 (C/T) | -6.06 | 0.28 | 0.014 | 0.87 | -0.050 | 0.54 | 0.036 | 0.27 | 0.007 | 0.50 |
| *BCDIN3D/FAIM2* | rs7138803 (A/G) | 0.25 | 0.96 | 0.014 | 0.64 | -0.043 | 0.57 | 0.030 | 0.18 | 0.011 | 0.57 |
| *SEC16B/RASAL2* | rs10913469 (C/T) | 0.32 | 0.80 | 0.009 | 0.75 | -0.031 | 0.71 | 0.022 | 0.42 | 0.037 | 0.21 |
| *BDNF* | rs4923461 (A/G) | -6.58 | 0.36 | -0.019 | 0.83 | -0.030 | 0.67 | 0.048 | 0.13 | 0.026 | 0.56 |
| *KCTD15/CHST8* | rs29941 (C/T) | 1.34 | 0.86 | -0.004 | 0.98 | 0.007 | 0.95 | -0.003 | 0.86 | 0.016 | 0.69 |
| *TMEM18* | rs6548238 (C/T) | -3.07 | 0.58 | -0.11 | 0.092 | 0.065 | 0.39 | 0.040 | 0.18 | 0.049 | 0.10 |
| *NCR3/A1F1/BAT2* | rs2844479 (T/G) | 8.85 | 0.040 | 0.051 | 0.33 | -0.055 | 0.35 | 0.004 | 0.76 | 0.011 | 0.57 |
| *PTER locus* | rs10508503 (C/T) | 0.26 | 0.88 | 0.085 | 0.35 | -0.074 | 0.41 | -0.011 | 0.76 | -0.045 | 0.14 |
| *MAF locus* | rs1424233 (A/G) | -5.42 | 0.17 | -0.079 | 0.20 | 0.12 | 0.020 | -0.040 | 0.042 | -0.011 | 0.97 |
| *NPC1* | rs1805081 (A/G) | -11.99 | 0.006 | -0.096 | 0.054 | 0.10 | 0.070 | -0.007 | 0.73 | 0.023 | 0.34 |

Abbreviations: SNP, single nucleotide polymorphism; (1/2)^a^, 1 refers to risk allele and 2 refers to other allele; β is the effect size associated with the risk allele. Adjusted for age, sex, season, diet assessment method and total energy intake when applicable. Log transformed variables were used e.g. lnTotal energy, lnFat etc.

**Table S4.** Interaction analysis between 16 obesity susceptibility SNPs and dietary intake levels on BMI, fat mass (FM) and fat free mass (FFM) among non-diabetic participants of MDCs cohort.

| BMI/obesity genes | P-Interaction | | | | | | | | | | | | | | |
| --- | --- | --- | --- | --- | --- | --- | --- | --- | --- | --- | --- | --- | --- | --- | --- |
|  | Total energy (kcal/day) | | | Fat (E%) | | | Carbohydrate (E%) | | | Protein (E%) | | | Fiber (g/1000kcal) | | |
|  | BMI  Βeta  (p) | FM  Βeta  (p) | FFM  Βeta  (p) | BMI  Βeta  (p) | FM  Βeta  (p) | FFM  Βeta  (p) | BMI  Βeta  (p) | FM  Βeta  (p) | FFM  Βeta  (p) | BMI  Βeta  (p) | FM  Βeta  (p) | FFM  Βeta  (p) | BMI  Βeta  (p) | FM  Βeta  (p) | FFM  Βeta  (p) |
| *FTO* | -0.025  (0.46) | -0.065  (0.53) | -0.022  (0.20) | 0.011  (0.58) | 0.072  (0.028) | 0.002  (0.98) | -0.027  (0.27) | -0.092  (0.013) | -0.001  (0.99) | 0.045  (0.14) | 0.067  (0.38) | 0.047  (0.22) | -0.003  (0.73) | -0.027  (0.20) | -0.011  (0.71) |
| *MC4R* | 0.039  (0.17) | -0.0003  (0.88) | 0.092  (0.26) | 0.010  (0.73) | -0.015  (0.79) | 0.044  (0.47) | -0.020  (0.49) | -0.017  (0.80) | -0.040  (0.45) | 0.023  (0.39) | 0.066  (0.20) | 0.026  (0.51) | 0.002  (0.84) | 0.042  (0.49) | -0.033  (0.72) |
| *SH2B1* | -0.013  (0.64) | -0.022  (0.90) | -0.019  (0.43) | 0.001  (0.87) | -0.005  (0.88) | -0.020  (0.64) | 0.004  (0.99) | -0.002  (0.80) | 0.052  (0.30) | -0.074  (0.004) | -0.079  (0.048) | -0.097  (0.074) | 0.001  (0.88) | 0.008  (0.81) | -0.005  (0.90) |
| *GNPDA2* | -0.059  (0.016) | -0.10  (0.017) | -0.015  (0.57) | -0.048  (0.027) | -0.064  (0.053) | -0.087  (0.019) | 0.054  (0.021) | 0.070  (0.059) | 0.086  (0.033) | -0.008  (0.89) | -0.017  (0.92) | 0.011  (0.58) | 0.035  (0.093) | 0.080  (0.014) | 0.025  (0.44) |
| *MTCH2* | 0.051  (0.032) | 0.080  (0.028) | 0.053  (0.23) | 0.054  (0.022) | 0.097  (0.020) | 0.037  (0.36) | -0.067  (0.007) | -0.12  (0.004) | -0.064  (0.14) | 0.005  (1.00) | 0.038  (0.51) | 0.017  (0.56) | -0.016  (0.57) | -0.018  (0.92) | 0.028  (0.49) |
| *NEGRI* | -0.026  (0.38) | -0.039  (0.67) | -0.001  (0.93) | 0.031  (0.20) | 0.061  (0.15) | 0.065  (0.10) | -0.013  (0.60) | -0.036  (0.38) | -0.031  (0.41) | -0.002  (0.95) | -0.009  (0.77) | -0.014  (0.78) | -0.007  (0.79) | -0.003  (0.87) | -0.019  (0.65) |
| *SFRS10* | 0.025  (0.52) | 0.003  (0.59) | 0.037  (0.74) | 0.013  (0.68) | 0.006  (0.83) | 0.080  (0.12) | -0.036  (0.25) | -0.048  (0.29) | -0.14  (0.005) | 0.005  (0.90) | 0.034  (0.55) | 0.020  (0.55) | 0.010  (0.68) | -0.018  (0.85) | -0.051  (0.57) |
| *BCDIN3D/FAIM2* | -0.021  (0.41) | -0.031  (0.53) | -0.070  (0.055) | -0.023  (0.36) | -0.051  (0.17) | 0.009  (0.79) | 0.028  (0.23) | 0.050  (0.13) | -0.001  (0.93) | 0.007  (0.91) | 0.003  (0.83) | 0.003  (0.96) | 0.015  (0.63) | 0.035  (0.43) | -0.020  (0.65) |
| *SEC16B/RASAL2* | -0.022  (0.42) | -0.059  (0.14) | -0.001  (0.84) | -0.010  (0.82) | 0.007  (0.89) | -0.026  (0.76) | 0.004  (0.94) | -0.017  (0.76) | 0.030  (0.66) | 0.011  (0.75) | 0.011  (0.74) | -0.013  (0.80) | -0.055  (0.054) | -0.068  (0.23) | -0.16  (0.003) |
| *BDNF* | -0.018  (0.63) | -0.058  (0.36) | 0.074  (0.27) | -0.016  (0.68) | -0.006  (0.87) | -0.009  (0.90) | -0.006  (0.87) | -0.016  (0.67) | -0.003  (1.00) | 0.022  (0.001) | 0.039  (0.66) | -0.080  (0.11) | -0.011  (0.51) | -0.001  (0.58) | -0.031  (0.50) |
| *KCTD15/CHST8* | 0.003  (0.91) | -0.11  (0.73) | 0.013  (0.83) | 0.004  (0.93) | -0.025  (0.47) | 0.002  (0.93) | -0.015  (0.58) | 0.006  (0.80) | -0.035  (0.57) | 0.032  (0.22) | 0.060  (0.13) | 0.071  (0.15) | -0.027  (0.27) | -0.029  (0.43) | -0.033  (0.45) |
| *TMEM18* | 0.0001  (0.040) | 0.0001  (0.11) | 1.9x10^-5^  (0.83) | -0.013  (0.61) | -0.027  (0.61) | -0.016  (0.61) | 0.010  (073) | 0.014  (0.89) | -0.006  (0.92) | 0.045  (0.22) | 0.073  (0.22) | 0.11  (0.055) | -0.016  (0.64) | 0.005  (0.77) | -0.001  (0.86) |
| *NCR3/A1F1/BAT2* | 8.7x10^-5^  (0.11) | 0.0001  (0.089) | 0.0002  (0.16) | -0.018  (0.61) | -0.054  (0.42) | -0.039  (0.38) | 0.026  (0.35) | 0.059  (0.26) | 0.037  (0.43) | 0.001  (0.92) | 0.003  (0.72) | 0.038  (0.33) | -0.018  (0.28) | -0.011  (0.36) | -0.031  (0.69) |
| *PTER locus* | 6.8x10^-5^  (0.49) | 3.4x10^-5^  (0.76) | -0.0001  (0.56) | 0.017  (0.85) | 0.018  (1.00) | 0.059  (0.49) | 0.003  (0.72) | 0.027  (0.42) | -0.073  (0.46) | -0.093  (0.014) | -0.17  (0.003) | 0.031  (0.96) | -0.041  (0.36) | -0.009  (0.70) | -0.032  (0.81) |
| *MAF locus* | 2.1x10^-5^  (0.62) | -1.2x10^-5^  (0.97) | 5.2x10^-5^  (0.43) | 0.049  (0.044) | 0.063  (0.21) | 0.072  (0.077) | -0.061  (0.011) | -0.083  (0.065) | -0.049  (0.25) | 0.010  (0.72) | 0.023  (0.57) | -0.031  (0.34) | -0.023  (0.36) | -0.022  (0.73) | -0.050  (0.27) |
| *NPC1* | -5.6x10^-5^  (0.31) | -0.0001  (0.46) | -5.8x10^-6^  (0.67) | 0.0005  (0.94) | 0.002  (0.79) | -0.024  (0.52) | -0.014  (0.55) | -0.005  (0.75) | -0.019  (0.73) | 0.053  (0.023) | 0.042  (0.32) | 0.077  (0.037) | 0.014  (0.56) | 0.030  (0.58) | 0.018  (0.45) |

Abbreviations: BMI, body mass index; FM, fat mass; FFM, fat free mass. β indicates the magnitude of the difference between the associations with BMI per risk allele and quintile of dietary intakes.  Positive values indicate that the increase in BMI by the risk allele becomes more pronounced  at high dietary intakes, whereas negative values indicate a more pronounced  increase in BMI by the risk allele at low dietary intakes Adjusted for age, se. x, season, diet assessment method and total energy intake when applicable. P-Interaction values were obtained using ln transformed variables i-e lnBMI, lnFM and lnFFM.

**Supplementary Figures**


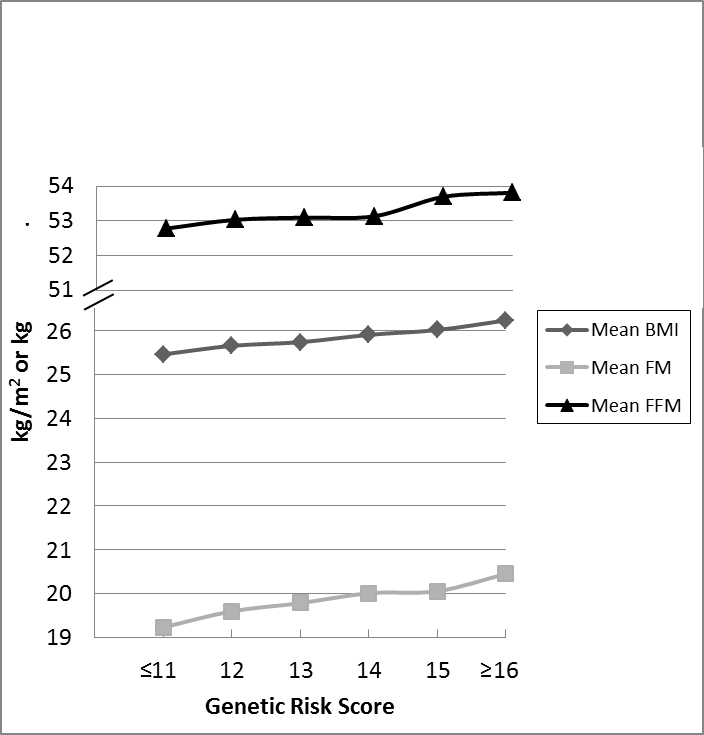


**Fig 2** Mean BMI, fat mass (FM) and fat-free mass (FFM) by the number of risk alleles of the genetic risk score (GRS) in the Malmö Diet and Cancer Study.


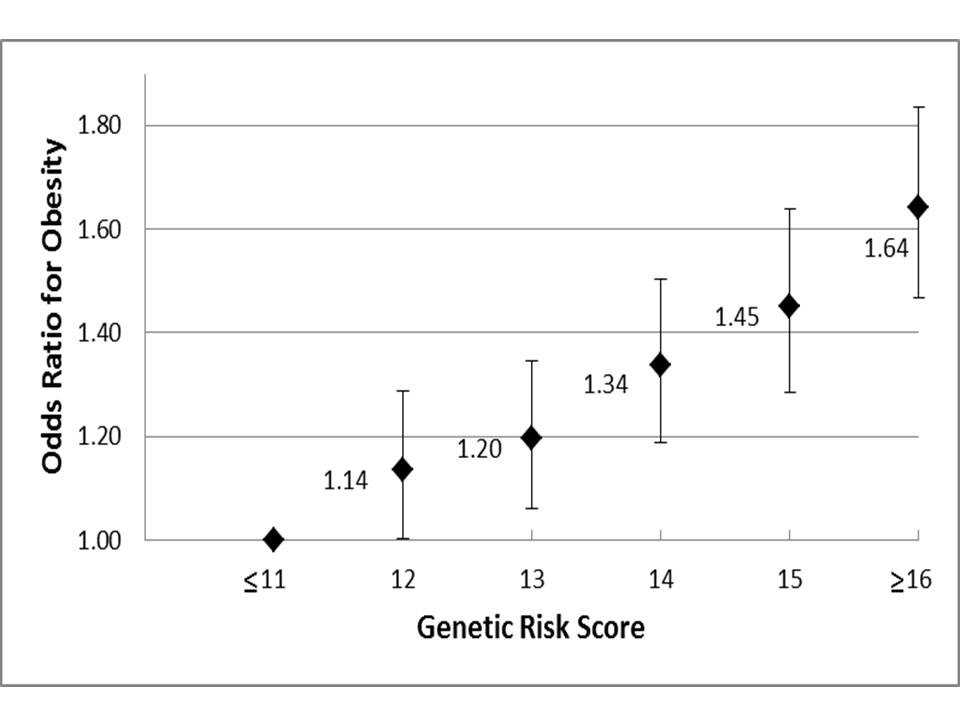


**Fig 3** Relationship between the number of risk alleles of the genetic risk score (GRS) and the risk of being obese in the Malmö Diet and Cancer Study. The graph shows odds ratios indicated as diamonds with increasing GRS as compared to the reference group with GRS of ≤11. The 95% confidence intervals are represented as error bars.


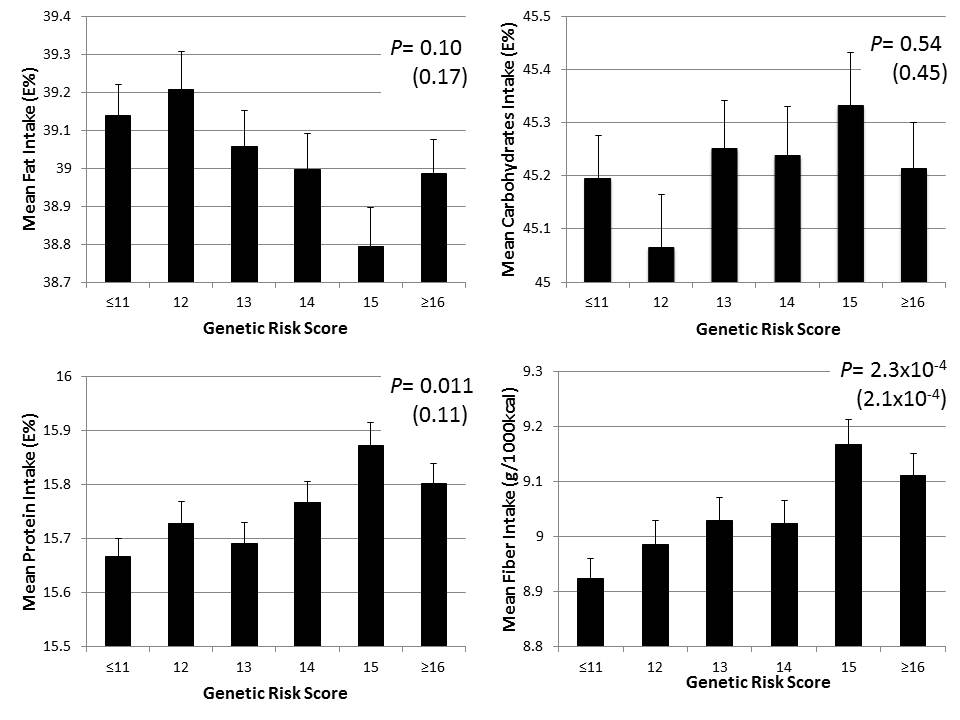


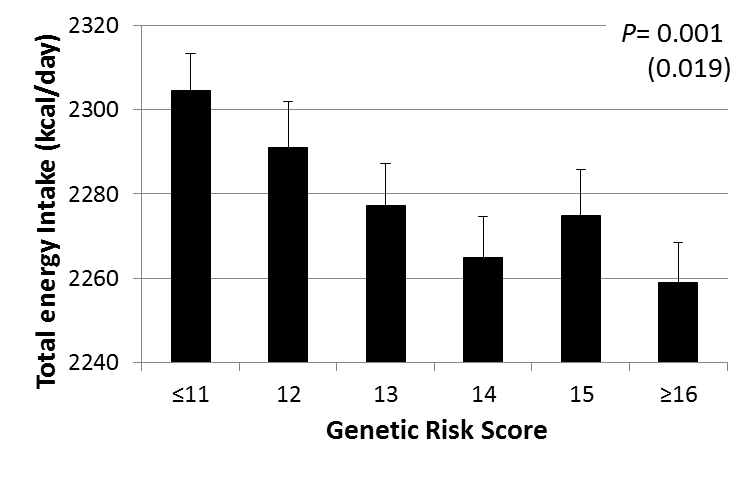


**Fig 4** Mean intakes of macronutrients (measured as energy %), fiber (measured as g/1000kcal) and total energy intake (measured as kcal/day) according to increasing genetic risk score (GRS) among 26107 non-diabetic participants of the Malmö Diet and Cancer Study. Values on the y-axis represent the mean intakes of total energy, macronutrients and fiber. Values on the x-axis represent the number of risk alleles in each group of GRS. *P* values are calculated after adjusting for age, sex, season, diet assessment method version and total energy intake when applicable. P values after excluding misreporters are shown in parenthesis.


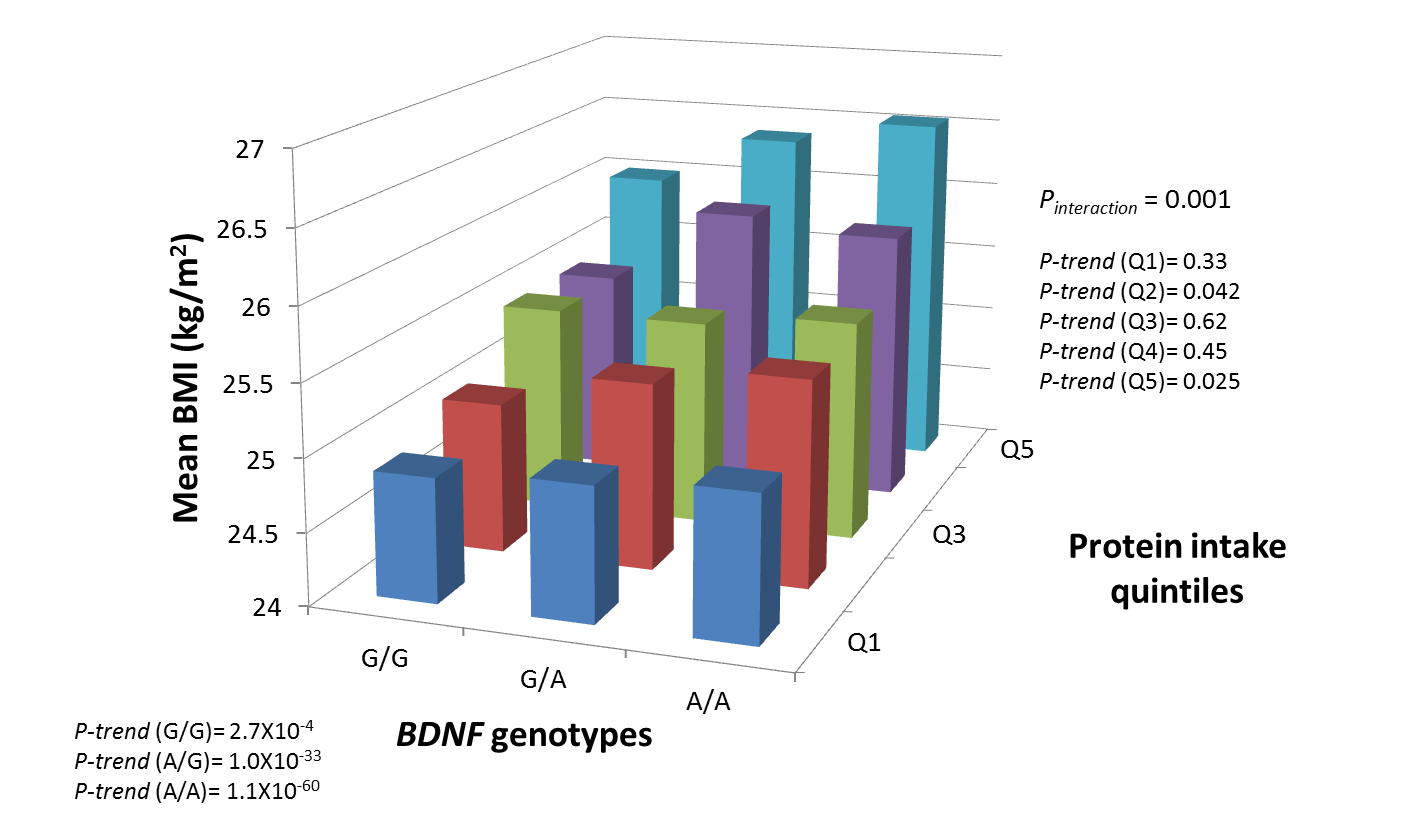


**Fig 5** Association between *BDNF* rs4923461 genotype and BMI in strata of protein intake quintiles (measured as energy %) in the Malmö Diet and Cancer Study. Linear regression was used to calculate P-trend adjusted for age, sex, season, diet assessment method and total energy intake. The interaction between *BDNF* genotypes and protein intake quintiles on BMI was assessed by introducing a multiplicative factor with continuous variables.
